# Production of Ethyl-agarobioside, a Novel Skin Moisturizer, by Mimicking the Alcoholysis from the Japanese Sake-Brewing Process

**DOI:** 10.3390/md21060341

**Published:** 2023-06-01

**Authors:** Sun-Hee Lee, Eun Ju Yun, Na Ree Han, Inho Jung, Jeffrey G. Pelton, Jae-Eun Lee, Nam Joo Kang, Yong-Su Jin, Kyoung Heon Kim

**Affiliations:** 1 Department of Biotechnology, Graduate School, Korea University, Seoul 02841, Republic of Korea; wowshl@korea.ac.kr (S.-H.L.); nareehan@korea.ac.kr (N.R.H.); 2 Division of Biotechnology, Jeonbuk National University, Iksan 54596, Republic of Korea; ejyun@jbnu.ac.kr; 3 Korea Forestry Promotion Institute, Daejeon 34215, Republic of Korea; wind9887@hanmail.net; 4 QB3 Institute, University of California, Berkeley, CA 94720, USA; jgpelton@berkeley.edu; 5 School of Food Science and Biotechnology, Kyungpook National University, Daegu 41566, Republic of Korea; lju1033@naver.com; 6 Department of Biotechnology, The Catholic University of Korea, Bucheon 14662, Republic of Korea; 7 Department of Food Science and Human Nutrition, University of Illinois at Urbana-Champaign, Urbana, IL 61801, USA

**Keywords:** ethyl-agarobioside, agarobiose, alcoholysis, agarose, red seaweed, moisturizer

## Abstract

Agarobiose (AB; d-galactose-β-1,4-AHG), produced by one-step acid hydrolysis of agarose of red seaweed, is considered a promising cosmetic ingredient due to its skin-moisturizing activity. In this study, the use of AB as a cosmetic ingredient was found to be hampered due to its instability at high temperature and alkaline pH. Therefore, to increase the chemical stability of AB, we devised a novel process to synthesize ethyl-agarobioside (ethyl-AB) from the acid-catalyzed alcoholysis of agarose. This process mimics the generation of ethyl α-glucoside and glyceryl α-glucoside by alcoholysis in the presence of ethanol and glycerol during the traditional Japanese sake-brewing process. Ethyl-AB also showed in vitro skin-moisturizing activity similar to that of AB, but showed higher thermal and pH stability than AB. This is the first report of ethyl-AB, a novel compound produced from red seaweed, as a functional cosmetic ingredient with high chemical stability.

## 1. Introduction

Seaweeds contain abundant polysaccharides that have various physiological functions [1,2,3,4,5]. For example, agar, the primary cell wall matrix of red seaweeds, is composed of agarose and porphyran (charged agarose) [6,7].

Agarose is a unique heteropolysaccharide consisting of d-galactose and 3,6-anhydro-l-galactose (AHG), which are linked by alternating α-1,3- and β-1,4-glycosidic bonds [8,9]. Agarose can be hydrolyzed into agaro-oligosaccharides (AOSs; oligosaccharides with galactose at the non-reducing end) and neoagaro-oligosaccharides (NAOSs; oligosaccharides with AHG at the non-reducing end) by cleaving α-1,3- and β-1,4-glycosidic bonds, respectively (Figure S1) [10,11,12]. In particular, AHG and AHG-containing oligosaccharides have been shown to have functional cosmetic effects, such as skin-whitening and moisturizing activities [13,14].

Recently, simple one- or two-step processes have been developed to produce agarobiose (AB; d-galactose-β-1,4-AHG) or AHG from agar [15]. These processes may pave the way for the mass production of these specialty sugars as functional food and cosmetic ingredients. However, because of the high reactivity of the aldehyde group at the C1 position of AHG [11], AHG or AB can be unstable under extreme states, such as high temperatures or extreme pH conditions [11,16,17]. For a functional material to be used as a cosmetic ingredient, it has to withstand temperatures over 45 °C for more than four weeks and be stable in the pH range of 3–9, depending on the cosmetic product type [18,19,20]. For example, AHG is unstable under acidic conditions and easily degrades into 5-hydroxymethylfurfural (5-HMF) and other byproducts, such as levulinic acid and formic acid [11,17]. For the industrial use of functional cosmetic ingredients, higher pH stability of the ingredient in the pH range of 3–9 is required. For example, cosmetic products containing α-hydroxy acids are recommended to have a pH ≥ 3.0 at the final formulation [19]. The recommended pH ranges for antiseptic cleansers and mild cleansers are pH 8.9–9.6 and pH 6.9–7.5, respectively [20].

Hyaluronic acid (HA) is a key molecule in skin moisture and is synthesized by three HA synthases (HASs), HAS-1, HAS-2, and HAS-3, which have different catalytic activity (HAS-1 < HAS-2 < HAS-3) [21,22]. HAS-2 has a critical role mainly in human normal cells, but HAS-3 has a role in tumor cells predominantly [23]. HAS-2 can be the most highly stimulated isoform in keratinocytes, leading to increasing epidermal HA [21,23].

In this study, because AB can be directly produced on a large scale from agar by acid hydrolysis and is presumed to be biologically functional, such as AHG, chemical modification of AB was sought to increase its stability under extreme conditions (high temperature and pH) to develop AB as a functional cosmetic ingredient. The generation of ethylated glucoside during the traditional Japanese sake-brewing process was adopted as a concept for the chemical modification of AB. Ethylated sugars, mostly ethyl α-d-glucoside (α-EG), are easily produced during the sake-brewing process via alcoholysis of starch in the presence of ethanol, instead of hydrolysis, by fungal hydrolases [24]. In addition, α-EG has been shown to have protective activities against skin roughness after ultraviolet B irradiation, transepidermal water loss, and hepatic disorder [25].

In this study, we designed the acid-catalyzed alcoholysis of agarose in which the α-1,4-glycosidic linkages of agarose are preferentially cleaved [15], thus generating ethyl-agarobioside (ethyl-AB) as the major product. Since ethyl-AB is a novel compound, its chemical structure was analyzed using mass spectrometry (MS) and nuclear magnetic resonance (NMR). Subsequently, we tested the in vitro cytotoxicity and moisturizing activities of AB and ethyl-AB on immortalized human epidermal keratinocyte (HaCaT) cells and their thermal and pH stability.

To the best of our knowledge, this is the first study on the production of ethyl-AB via a novel one-step acid-catalyzed alcoholysis of agarose and the functional evaluation of ethyl-AB as a cosmetic ingredient by demonstrating their in vitro moisturizing activities. We envision that ethyl-AB can be widely used as high-value cosmetic ingredients derived from red seaweeds.

## 2. Results and Discussion

### 2.1. Skin-Moisturizing Activity of AB

**Figure 1:**
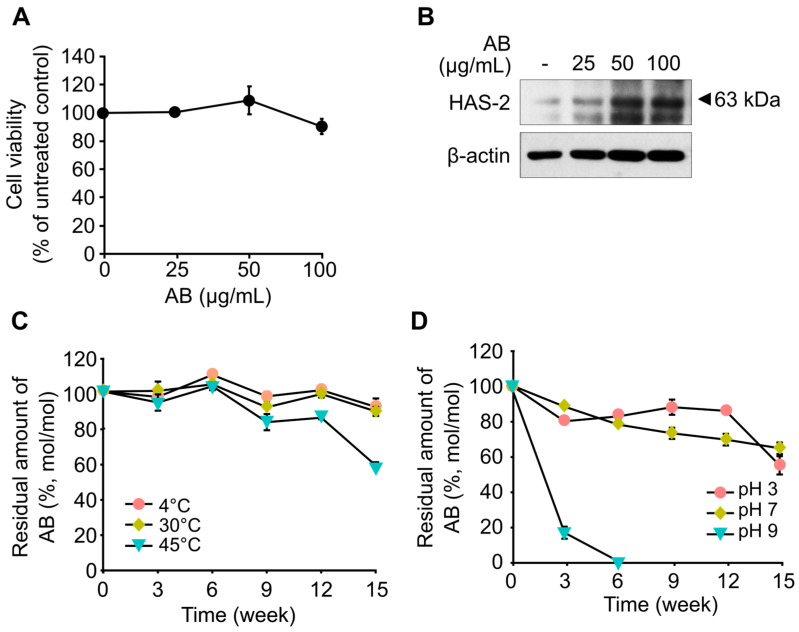
In vitro skin-moisturizing activity and stability of AB. (**A**) Cytotoxic effects of the indicated AB concentrations on HaCaT cells as determined by MTT assay. (**B**) Effect of AB on HAS-2 expression in HaCaT cells. AB (25, 50, or 100 μg/mL) was used to treat cells starved in serum-free DMEM for 6 h. HAS-2 and β-actin expression levels in cell lysates were determined by Western blot. (**C**) Thermal stability of AB at various incubation conditions. AB was incubated at 4, 30, and 45 °C for 15 weeks. All data represent the means and standard deviations of three independent replicates. (**D**) pH stability of AB at various incubation conditions. AB was incubated at pH 3 in 20 mM citrate at 25 °C for 15 weeks and at pH 7 and 9 in 20 mM of Tris-HCI at 25 °C for 15 weeks. All data represent the means and standard deviations of three independent replicates.

Prior to investigating the skin-moisturizing activity of AB, its cytotoxicity against HaCaT cells was evaluated (Figure 1A). The results showed that up to 100 μg/mL of AB did not exhibit cytotoxicity to HaCaT cells for 24 h (Figure 1A). To investigate the in vitro skin-moisturizing activity of AB, the effects of AB treatment on the expression of HAS-2 in HaCaT cells were measured. The results revealed that HAS-2 expression increased in a dose-dependent manner following AB treatment (Figure 1B). AHG, which is the monomeric sugar at the reducing end of AB, was also shown to exhibit skin-moisturizing activity in vitro, similar to the findings in our previous study [26]. The moisturizing activity of AHG was found to induce the upregulation of hyaluronic acid synthesis in a dose-dependent manner by increasing HAS-2 expression [26]. In addition, AHG was found to activate the AMPKα signaling pathway, which regulates HAS-2 expression in HaCaT cells [26].

Similarly, the moisturizing effect of neoagarobiose (NeoDP2) was previously observed in vitro [27], but the production of NeoDP2 is much more complex than that of AB, consisting of a multi-step process to hydrolyze agarose using strong acids and enzymes [15]. Unlike NeoDP2, AB can be produced by single-step acid hydrolysis of agarose [15]. Under acid hydrolysis conditions, the cleavage of α-glycosidic linkages of agarose is preferred to that of its β-glycosidic linkages [12]; thus, AB is easily produced from the weak acid hydrolysis of agarose or agar [11,28]. Therefore, the in vitro skin-moisturizing activity of AB, verified by the induction of HAS-2 expression in HaCaT cells, is promising because the mass production of AB as a skin moisturizing agent is technically possible [15].

### 2.2. Thermal and pH Stabilities of AB

Because functional cosmetic ingredients should have thermal and pH stabilities, the thermal and pH stabilities of AB were verified. First, the thermal stability of AB was verified by monitoring the residual amount of AB after incubation at 4, 30, and 45 °C, for 15 weeks without pH control (Figure 1C). The results showed that more than 80% of AB was stably maintained, regardless of the incubation temperature, until 12 weeks (Figure 1C). However, at 45 °C, the residual amount of AB decreased to 58% of its initial amount after 15 weeks (Figure 1C). In our previous study, AHG showed low thermal stability at incubating temperatures of 50 °C or higher. At 50 °C, AHG begins to degrade from the initial period and decreases to less than 10% of its initial amount after 15 weeks [16]. Therefore, AB can be considered more stable than AHG, but it is still relatively unstable at high temperatures.

Next, the pH stability of AB was evaluated by monitoring the residual amount of AB after incubation at pH 3, 7, and 9 for 15 weeks at 25 °C. At pH 3, more than 80% of AB remained until 12 weeks, but the residual amount of AB significantly decreased to 54% of the initial amount after 15 weeks (Figure 1D). At pH 7, AB gradually decreased during incubation, and the residual amount of AB after 15 weeks was 64% (Figure 1D). Notably, at pH 9, AB was highly unstable, and AHG was completely degraded within six weeks (Figure 1D).

In addition to our aforementioned findings of the in vitro skin-moisturizing activity of AB, antioxidant and anti-inflammatory activities of AB also have been demonstrated [29,30]. However, the use of AB as a functional material, especially as a cosmetic ingredient, is challenging due to the instability of AB, especially at high temperatures (i.e., >45 °C) or at alkaline pH (i.e., pH > 9).

### 2.3. Production of Ethyl-AB by Acid-Catalyzed Alcoholysis of Agarose

**Figure 2:**
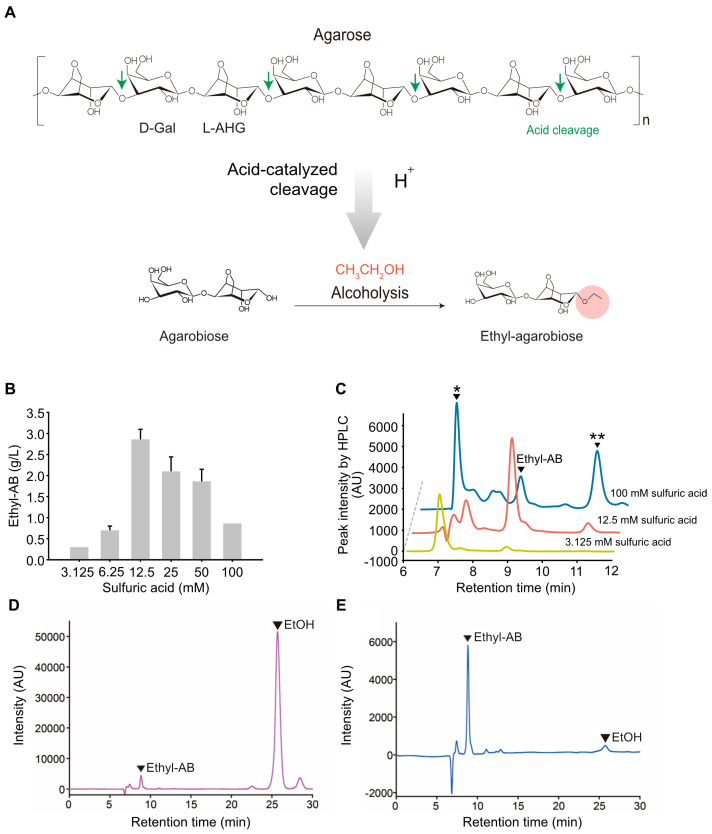
Acid-catalyzed alcoholysis and purification for ethyl-AB production. (**A**) Chemical structures and the reaction scheme of acid-catalyzed alcoholysis of agarose. The produced ethyl-AB represents the basic unit of AOSs released by acid-catalyzed alcoholysis. (**B**) Alcoholysis was performed on 2% (*w/v*) of agarose in ethanol at 70 °C overnight using various concentrations of sulfuric acid. (**C**) Reaction products of acid-catalyzed alcoholysis of agarose into ethyl-AB as analyzed by HPLC. * Peak representing oligomers above DP4 from agarose depolymerization and sulfuric acid. ** Peak representing ethyl-3,6-anhydro-galactoside and ethyl-galactoside, which have the same retention time under the specified HPLC conditions. (**D**) HPLC chromatogram of the alcoholysis products before ethanol removal by vacuum evaporation. (**E**) HPLC chromatogram of the collected ethyl-AB fractions purified from the vacuum-evaporated concentrate and separated by size-exclusion chromatography using a Sephadex G-10 column.

The instability of AB is probably due to AHG, which is located at the reducing end of AB. AHG is known to be unstable and easily degrades into 5-HMF by dehydration at high temperatures [31]. Therefore, to improve the chemical stability of AB, we attempted to chemically modify AB using the acid-catalyzed alcoholysis of agarose, which produces ethyl-AB (Figure 2A).

During the brewing process of sake, a Japanese rice wine, a variety of oligosaccharides and glucosides are produced from starch by the hydrolysis and transglycosylation activity of fungal α-glucosidases [32]. In particular, ethyl α-glucoside (α-EG) and glyceryl α-glucoside (α-GG) are generated by alcoholysis catalyzed by fungal α-glucosidases in the presence of ethanol and glycerol as acceptor molecules, respectively [33,34,35,36]. Owing to their skin moisturizing effects, α-EG or α-GG byproducts from sake brewing are used as cosmetic ingredients [37,38]. Their moisturizing effects could explain the traditional concept of sake brewers having fair and smooth hands. Therefore, in this study, alcoholysis from the sake brewing process was adopted for the acid-catalyzed alcoholysis of agarose to produce ethyl-AB.

Various acid catalysts such as sulfuric, hydrochloric, nitric, and phosphoric acids, were tested for the acid-catalyzed hydrolysis of agarose to produce ethyl-AB. Among the various acid catalysts, sulfuric acid and hydrochloric acid produced ethyl-AB most efficiently (Figure S2). The optimal concentration of sulfuric acid was determined based on the amount of ethyl-AB in the reaction mixture (Figure 2B). The results showed that among various concentrations (3.125, 12.5, and 100 mM) of sulfuric acid, 12.5 mM was the optimal concentration for producing ethyl-AB (Figure 2B). With 3 mM sulfuric acid, reaction products with the degrees of polymerization (DPs) higher than DP2 were produced due to the insufficient breakdown of α-1,3-glycosidic bonds of agarose (Figure 2C). With 100 mM sulfuric acid, ethyl-AB was further degraded to ethyl-galactoside and 3,6-anhydro-ethyl-galactoside (Figure 2C).

Ethanol was removed from the reaction mixture (Figure 2D) using a speed vacuum evaporator, which simultaneously concentrated the mixture. Ethyl-AB in the concentrate was then separated from sulfuric acid and other byproducts by gel filtration chromatography using a Sephadex G-10 column, and the fractions containing only ethyl-AB were collected and analyzed as a single major peak by HPLC (Figure 2E).

### 2.4. Identification of Ethyl-AB by MS and NMR Analyses

**Figure 3:**
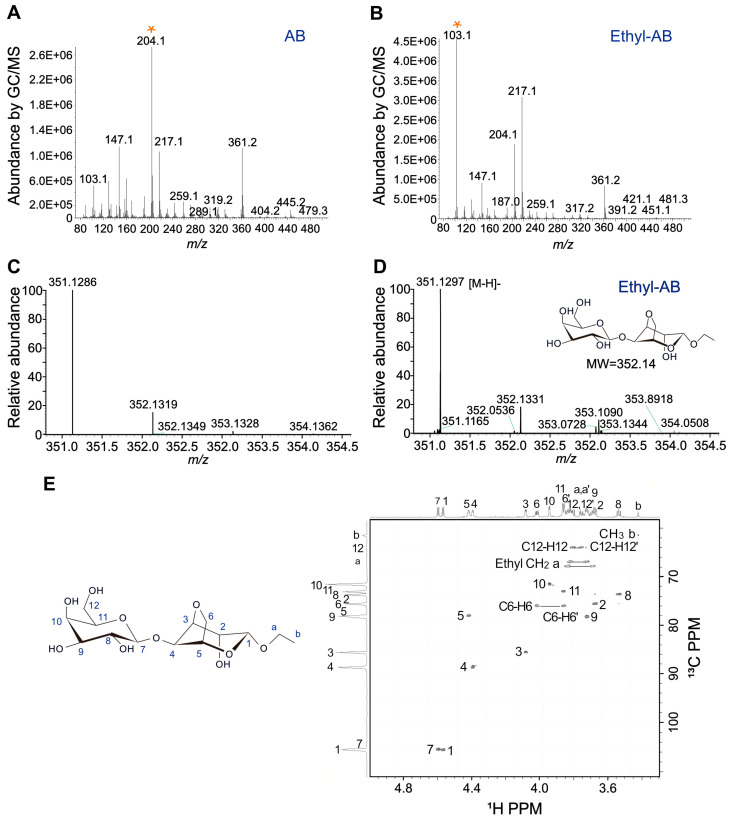
Structural identification of ethyl-AB. The mass spectra of (**A**) AB and (**B**) ethyl-AB with the characteristic mass fragmentation patterns with unique ion peaks of AB and ethyl-AB indicated by * were obtained using GC/MS analysis. (**C**) Theoretical and (**D**) experimental mass spectra of ethyl-AB with its indicated molecular weight identified using LC/HRMS: the theoretical major peak at *m/z* of 351.1286 and the experimental major peak at *m/z* of 351.1297 with error of 3.13 ppm. The separations were carried out using liquid chromatography and analyzed using a Q-Exactive orbitrap high resolution mass spectrometer. (**E**) ^1^H-^13^C HSQC NMR spectrum of ethyl-AB.

Since ethyl-AB is a novel compound synthesized in this study, structural identification of ethyl-AB was performed by MS and NMR analyses. First, the mass spectra obtained from GC/MS analyses of AB and ethyl-AB were compared (Figure 3A,B). Some unique fragment ions of AB and ethyl-AB overlapped, likely due to the structural similarity between AB and ethyl-AB. The overall mass fragmentation patterns between AB and ethyl-AB in the mass spectra were different (Figure 3A,B).

MS analysis was performed to determine the exact mass of ethyl-AB. In negative ion mode, ethyl-AB showed major peaks at *m/z* of 351.1286 and 351.1297 in the theoretical and experimental mass spectra, respectively, which corresponded to the exact mass of deprotonated ethyl-AB (Figure 3C,D). Thus, the dominant peak was assigned to ethyl-AB, a dimer with a molecular mass of 352.14, composed of galactose and 3,6-anhydro-ethyl-galactoside (Figure 3D).

After obtaining the exact mass of ethyl-AB, its chemical structure was identified using NMR analysis. The ^1^H–^13^C heteronuclear single quantum coherence (HSQC) spectrum of ethyl-AB showed that all the signals obtained matched well with those of ethyl-AB (Figure 3E). In particular, the two protons at the C1-position of ethyl-AB indicated that the aldehyde group at C1 did not exist in ethyl-AB (Figure 3E). Instead, we identified an ethyl group by measuring the CH_2_ and CH_3_ signals in ^1^H–^13^C HSQC spectrum of ethyl-AB (Figure 3E). Two different signals were observed for the CH_2_ group of ethyl-AB. Therefore, we suggested that acid-catalyzed alcoholysis of agarose produces ethyl-AB with the two different anomeric forms, α- and β-forms [39]. These results indicated that the aldehyde group at the 1-position was converted into an ethyl group.

### 2.5. Skin-Moisturizing Activity of Ethyl-AB

**Figure 4:**
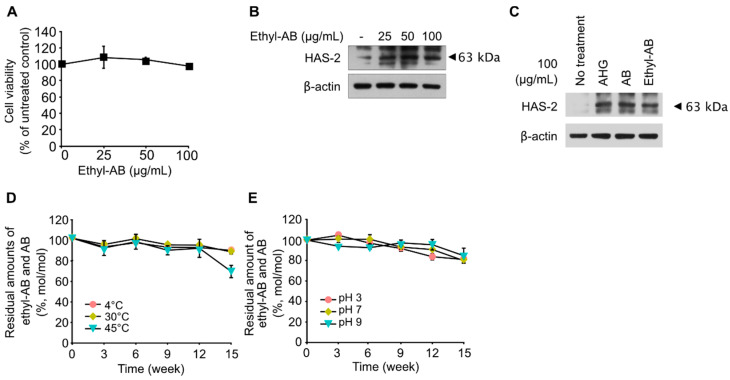
In vitro skin-moisturizing activity and thermal and pH stabilities of ethyl-AB. (**A**) Cytotoxic effects of the indicated ethyl-AB concentrations on HaCaT cells as determined by MTT assay. (**B**) Effect of ethyl-AB on HAS-2 production in HaCaT cells. Cells were treated with ethyl-AB (25, 50, or 100 μg/mL) in serum-free DMEM for 6 h. HAS-2 and β-actin expression in cell lysates were determined using Western blot. (**C**) Effects of AHG, AB, and ethyl-AB on HAS-2 expression. Cells were starved in serum-free DMEM and treated with 100 μg/mL AHG, AB, or ethyl-AB. HAS-2 and β-actin expression levels in cell lysates were determined by Western blot. (**D**) Thermal stability of ethyl-AB incubated at 4, 30, and 45 °C for 15 weeks. The values and error bars represent the means and standard deviations of three independent replicates. (**E**) pH stability of ethyl-AB incubated at 25 °C in 20 mM citrate (pH 3) and in 20 mM Tris-HCI (pH 7 and 9) for 15 weeks. Data points represent the means and standard deviations of three independent replicates.

Before evaluating the skin-moisturizing activity of ethyl-AB, the cytotoxicity of ethyl-AB was examined through proliferation assays using HaCaT cells. Similar to AB, ethyl-AB also did not inhibit the growth of HaCaT cells for 24 h (Figure 4A). To investigate the in vitro skin-moisturizing activity of ethyl-AB, the effect of ethyl-AB on HAS-2 expression was evaluated in HaCaT cells treated with 0, 25, 50, and 100 μg/mL ethyl-AB, and the results indicated that HAS-2 expression in HaCaT cells was highly induced by ethyl-AB treatment in a dose-dependent manner (Figure 4B).

For comparative evaluation of the skin-moisturizing activity of AHG, AB and ethyl-AB, HaCaT cells were treated with 100 μg/mL of the three compounds. The increase in HAS-2 expression was similar in the AHG, AB, and ethyl-AB treatment groups (Figure 4C), probably because AHG, AB, and ethyl-AB have a high hygroscopic ability due to their deliquescent hydroxyl groups [27]. These results suggest that ethyl-AB can be used as a cosmetic skin moisturizer if it shows proper stability at relatively high temperatures and extreme pH values.

### 2.6. Thermal and pH Stabilities of Ethyl-AB

Since both AB and ethyl-AB exhibited similar in vitro skin moisturizing activity in this study, the thermal and pH stabilities of ethyl-AB were evaluated to evaluate its feasibility as a functional ingredient. Because both AB and ethyl-AB showed similar skin-moisturizing activities (Figure 4C) and most of the disappearing ethyl-AB was converted to AB (Figure S3), the combined amount of ethyl-AB and AB was monitored during the incubation period.

First, the thermal stability of ethyl-AB was tested after incubation at 4, 30, or 45 °C for 15 weeks without initial pH control (Figure 4D). For up to 12 weeks at 4 and 30 °C, 88.1 and 86.7% of ethyl-AB remained as ethyl-AB or AB, respectively (Figure 4D). At 45 °C, the residual amount of ethyl-AB or AB decreased to 67.7% of their initial amount after 15 weeks (Figure 4D), but it was higher than the residual amount of AB (i.e., 58%) when AB was incubated at the same temperature (Figure 1C). These results indicate that the initial form of ethyl-AB is more thermally stable as a cosmetic ingredient than AB.

Second, the pH stability of ethyl-AB was tested by monitoring its residual amount after incubation for 15 weeks at 25 °C and using an initial pH of 3, 7, or 9. After 15 weeks, 81, 80, and 84.8% of the initial ethyl-AB remained as residual ethyl-AB and AB at pH 3, 7, and 9, respectively (Figure 4E). Notably, ethyl-AB was stable at pH 9.0 (Figure 4E), while AB was completely degraded after six weeks (Figure 1D). These results indicate that ethyl-AB has a higher pH stability than AB.

Meanwhile, an ascorbic acid derivative, 3-O-ethyl-l-ascorbic acid (EA), exhibits efficient transdermal activity and is used in commercial cosmetics [40,41]. EA is a derivative of L-ascorbic acid that has an ethyl group at the C3 position [42]. The 3-O-ethyl derivative protects the 3-OH group from ionization and subsequent oxidation [42]. In the case of AB, the aldehyde group at the C1 position of the reducing end of AHG is converted into an ethyl group when AB is converted into ethyl-AB during alcoholysis of agarose. Therefore, similar to EA, ethyl-AB, an AB derivative with an ethyl group at the C1 position, can be used as a cosmetic ingredient with a higher pH stability and thermal tolerance than AB.

## 3. Materials and Methods

### 3.1. Preparation of AB and Ethyl-AB

AB was purified from commercial AOSs (Takara Bio, Shiga, Japan) produced by acid hydrolysis of agar by gel-permeation chromatography, using a GE Healthcare XK16/20 column (I.D. 16 × 200 cm; GE Healthcare, Buckinghamshire, UK) packed with Sephadex G-10 resin (GE Healthcare, Chicago, IL, USA). Water was used as the eluent at a flow rate of 0.3 mL/min. Fractions containing only AB, confirmed using high-performance liquid chromatography (HPLC), were collected to obtain high-purity AB.

To produce ethyl-AB, 2 g of agarose was added into 100 mL of 12.5 mM sulfuric acid in ethanol solution and incubated at 70 °C for 18 h for acid-catalyzed alcoholysis. After the reaction, the product was diluted with 300 mL water and neutralized with calcium hydroxide [43]. The precipitate was removed by centrifugation at 12,298× *g* for 15 min at room temperature, and the supernatant was collected. The residual precipitate in the supernatant was filtered through a membrane filter (pore size, 0.45 μm; Whatman, Dassel, Germany). The filtered supernatant containing mainly ethyl-AB was concentrated using a rotary vacuum evaporator at 35 °C for 4 h. The concentrated reaction product was purified using ÄKTA Prime system (GE Healthcare) equipped with a GE Healthcare XK16/20 column (I.D. 16 × 200 cm; GE Healthcare) packed with Sephadex G-10 resin (GE Healthcare). Water was used as the eluent at a flow rate of 0.3 mL/min. To obtain high-purity ethyl-AB, fractions containing ethyl-AB were collected and confirmed by HPLC analysis.

### 3.2. Analytical Methods

#### 3.2.1. HPLC Analysis

The concentrations of AB and ethyl-AB were measured using a HPLC system (Agilent 1100, Agilent Technologies, Wilmington, DE, USA) equipped with an Aminex HPX-87H column (Bio-Rad, Richmond, CA, USA). We set the temperatures of the column and refractive index detector to 65 °C and 55 °C, respectively. Sulfuric acid (5 mM) was used as the mobile phase at a flow rate of 0.5 mL/min.

#### 3.2.2. GC/MS Analysis

To analyze AB and ethyl-AB using gas chromatography/mass spectrometry (GC/MS), 5 μL each of the samples containing 1 g/L of AB or ethyl-AB was dried using a centrifugal vacuum evaporator (Labconco, Kansas, MO, USA). The dried samples were derivatized by adding 10 μL of 40 mg/mL methoxyamine chloride in pyridine (Sigma-Aldrich, St. Louis, MO, USA) and then incubating at 30 °C for 90 min. The samples were then subjected to trimethylsilylation by adding 45 μL of N-methyl-N-(tri-methylsilyl)-trifluoroacetamide (MSTFA; Fluka, St. Louis, MO, USA) and then incubating at 37 °C for 30 min.

The derivatized samples were analyzed using an Agilent 7890A GC/5975C MSD system (Agilent Technologies) with a RTX-5Sil MS column (30 m length, 0.25 mm inner diameter, and 0.25 µm film thickness; Restek, Bellefonte, PA, USA) with an additional 10-m guard column. The GC/MS operating conditions were as follows. The derivatized sample of 1 μL was injected into the GC column in splitless mode. The GC oven temperature was initially programmed at 50 °C for 1 min, and then it rapidly increased by 20 °C/min to 280 °C, and then it was held constant for 5 min. Electron ionization was performed at 70 eV. The temperatures of the ion source and transfer line were 250 °C and 280 °C, respectively. Mass spectra were recorded in the scan range of 80–700 *m/z*.

#### 3.2.3. LC/MS Analysis

Liquid chromatography/high resolution mass spectrometry (LC/HRMS) was used to analyze ethyl-AB. Direct-infusion measurements were carried out using a Q Exactive Orbitrap high-resolution mass spectrometer (Thermo Fisher Scientific, Bremen, Germany) with heated electrospray ionization (HESI) in negative ion mode. The samples were analyzed by HESI/MS with the following parameters: scan range of 350–450 *m/z*; sheath gas flow rate of 15 arbitrary units; spray voltage of −3.5 kV; capillary temperature of 320 °C; and S-lens RF level of 50 arbitrary units. The samples were injected at a rate of 5 μL/min into the HESI ion source using a syringe pump attached to the LC/HRMS system. Mass spectra acquisition and analysis were performed using Xcalibur software (version 4.0; Thermo Fisher Scientific, San Jose, CA, USA).

#### 3.2.4. NMR Analysis

Purified ethyl-AB was dried at 30 °C in a centrifugal vacuum concentrator and dissolved in deuterium oxide (D_2_O), and then it was subjected to nuclear magnetic resonance (NMR) analysis for the identification of ethyl-AB. ^1^H–^13^C NMR spectroscopy was performed using a 900 MHz NMR spectrometer equipped with a cryoprobe (Bruker Avance II; Bruker, Billerica, MA, USA). 3-(trimethylsilyl)-propionic-2,2,3,3-d_4_ acid (TSP) was used as a chemical shift reference.

### 3.3. Thermal and pH Stabilities of AB and Ethyl-AB

To test the thermal stability of AB and ethyl-AB, purified AB or ethyl-AB was dissolved in distilled water at a final concentration of 1 g/L without controlling the initial pH and incubated at 4, 30, or 45 °C for 15 weeks. To test the pH stability of AB and ethyl-AB, purified AB or ethyl-AB was dissolved in 20 mM citrate buffer (pH 3), 20 mM Tris-HCl buffer (pH 7), or 20 mM Tris-HCl buffer (pH 9) at a final concentration of 1 g/L and incubated at 25 °C for 15 weeks. We sampled every three weeks during incubation to monitor the concentrations of AB and ethyl-AB using HPLC and GC/MS analyses. All experiments were performed in triplicate.

### 3.4. In Vitro Skin-Moisturizing Activities of AB and Ethyl-AB

#### 3.4.1. Cell Proliferation Assay

To determine the cytotoxic effect of AB and ethyl-AB, a cell proliferation assay was performed. HaCaT cells were seeded in 96-well plates at a density of 3 × 10^4^ cells/well and cultured in Dulbecco’s modified Eagle’s medium (DMEM) supplemented with 10% (*v/v*) fetal bovine serum (FBS) in a 5% CO_2_ (*v/v*) incubator at 37 °C for 24 h. The HaCaT cells were treated with AB or ethyl-AB at a final concentration of 25, 50, or 100 μg/mL. After 6 h, 20 μL of 3-(4,5-dimethylthiazol-2-yl)-5-(3-carboxymethoxyphenyl)-2-(4-sulfonyl)-2 H-tetrazolium (MTT) solution was added to each well. The cells were then incubated for 2 h at 37 °C in a CO_2_ incubator, and the absorbance of the cell culture was measured at 570 nm using a microplate reader (Sunrise-Basic Tecan; Grodig, Austria). All experiments were performed in triplicate, and data are expressed as the mean ± standard deviation. For single statistical comparisons, Student’s *t*-test was performed with *p* < 0.05 considered statistically significant.

#### 3.4.2. In Vitro Skin-Moisturizing Activity

To evaluate the skin-moisturizing activity of AB and ethyl-AB, HaCaT cells were cultured at a density of 1 × 10^5^ cells/mL in a 60-mm dish for 24 h and then starved in serum-free DMEM. After a second 24 h period, the cells were treated with either AB, ethyl-AB, or AHG at the specified concentrations for 6 h. The cells were then harvested with a lysis buffer (20 mM Tris-HCl (pH 7.5), 150 mM NaCl, 1 mM ethylenediaminetetraacetic acid disodium salt, 1 mM ethylene glycol-bis(β-aminoethyl ether)-N,N,N′,N′-tetraacetic acid, 1% Triton X-100, 2.5 mM sodium pyrophosphate, 1 mM β-glycerophosphate, 1 mM Na_3_VO_4_, 1 μg/mL leupeptin, 1 mM phenylmethylsulfonyl fluoride, and a protease inhibitor cocktail tablet).

Protein concentration was determined using a dye-binding protein assay kit (Bio-Rad Laboratories, Hercules, CA, USA) according to the manufacturer’s instructions. Lysate protein (25 μg) was subjected to 10%-sodium dodecyl sulfate–polyacrylamide gel electrophoresis before being transferred into a polyvinylidene fluoride membrane (Millipore, Bedford, MA, USA). After blotting, the membrane was blocked with 5% skim milk for 2 h. The membrane was incubated with primary antibodies overnight at 4 °C, followed by secondary antibodies for 2 h, and the antibody-bound protein was labeled using a chemiluminescence detection kit (Amersham Pharmacia Biotech, Piscataway, NJ, USA). The expression levels of the hyaluronan synthase-2 (HAS-2) protein in each sample was detected by sensitizing the membranes to an X-ray film inside a dark room.

## 4. Conclusions

AB, which is considered a cosmetic skin moisturizer, was chemically modified to improve its thermal and pH stabilities. Ethyl-AB was produced by the acid-catalyzed alcoholysis of agarose, mimicking the generation of ethyl α-glucoside (α-EG) and glyceryl α-glucoside (α-GG) by alcoholysis of starch during the traditional Japanese sake-brewing process. Ethyl-AB was purified from the alcoholysis product mixture, and its chemical structure was analyzed using LC/HRMS and NMR. Ethyl-AB was found to possess skin-moisturizing activity similar to that of AB, whereas its thermal and pH stabilities were higher than that of AB. In particular, ethyl-AB showed high stability at pH 9, while AB was completely degraded after six weeks at the same pH. To our knowledge, this is the first report on the production of ethyl-AB and its skin-moisturizing activity as a novel functional cosmetic ingredient.

## Data Availability

Not applicable.

## Supplementary Materials

The following supporting information can be downloaded at: https://www.mdpi.com/article/10.3390/md21060341/s1, Figure S1: Structures of (A) agarooligosaccharides, (B) neoagarooligosaccharides, (C) agarobiose, (D) neoagarobiose, (E) 3,6-anhydro-l-galactose, and (F) d-galactose. Figure S2: HPLC analysis of the reaction products of agarose alcoholysis using various acid catalysts to produce ethyl-AB. Acid-catalyzed alcoholysis was performed on 2% (*w/w*) agarose at 70 °C for 18 h using 25 mM phosphoric acid, sulfuric acid, hydrochloric acid, or nitric acid as acid catalysts; Figure S3: Thermal stability of ethyl-AB and AB as measured by their residual amounts for 15 weeks at three different temperatures.
