# Supplementary material for: Production of Ethyl-agarobioside, a Novel Skin Moisturizer, by Mimicking the Alcoholysis from the Japanese Sake-Brewing Process
Source: Mar Drugs. 2023 Jun 1;21(6):341. doi: 10.3390/md21060341 (PMC10305320; doi:10.3390/md21060341)
Supplement: Supplementary file 1 [file marinedrugs-21-00341-s001.zip › marinedrugs-2376642-supplementary.pdf]

Supplementary Information

Production of Ethyl-agarobioside, a Novel Skin Moisturizer, by Mimicking the Alcoholysis from the Japanese Sake-Brewing Process

Sun-Hee Lee 1,†, Eun Ju Yun 2,†, Na Ree Han 1, Inho Jung 3, Jeffrey G. Pelton 4, Jae-Eun Lee 5, Nam Joo Kang 6,*, Yong-Su Jin 7,* and Kyoung Heon Kim 1,*

1 Department of Biotechnology, Graduate School, Korea University, Seoul 02841, Republic of Korea; wowshl@korea.ac.kr (S.-H.L.); nareehan@korea.ac.kr (N.R.H.)

2 Division of Biotechnology, Jeonbuk National University, Iksan 54596, Republic of Korea; ejyun@jbnu.ac.kr

3 Korea Forestry Promotion Institute, Daejeon 34215, Republic of Korea; wind9887@hanmail.net

4 QB3 Institute, University of California, Berkeley, CA 94720, USA; jgpelton@berkeley.edu

5 School of Food Science and Biotechnology, Kyungpook National University, Daegu 41566, Republic of Korea; lju1033@naver.com

6 Department of Biotechnology, The Catholic University of Korea, Bucheon 14662, Republic of Korea

7 Department of Food Science and Human Nutrition, University of Illinois at Urbana-Champaign, Urbana, IL 61801, USA

* Corresponding authors: njkang@catholic.ac.kr (N.J.K.); ysjin@illinois.edu (Y.-S.J.); khekim@korea.ac.kr (K.H.K.)

† These authors contributed equally to this work.

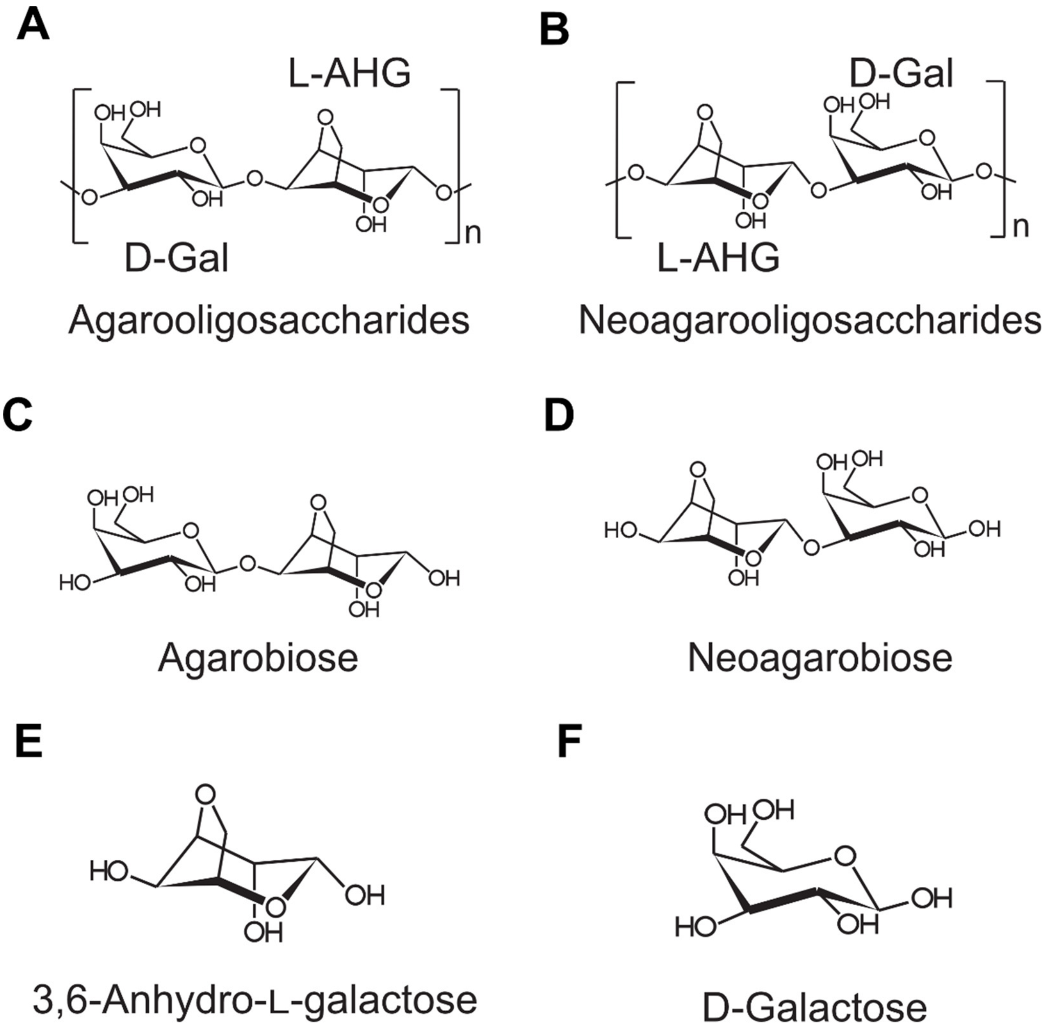

Figure S1. Chemical Structures of **(A)** agarooligosaccharides, **(B)** neoagarooligosaccharides, **(C)** agarobiose, **(D)** neoagarobiose, **(E)** 3,6-anhydro-L-galactose, and **(F)** D-galactose.

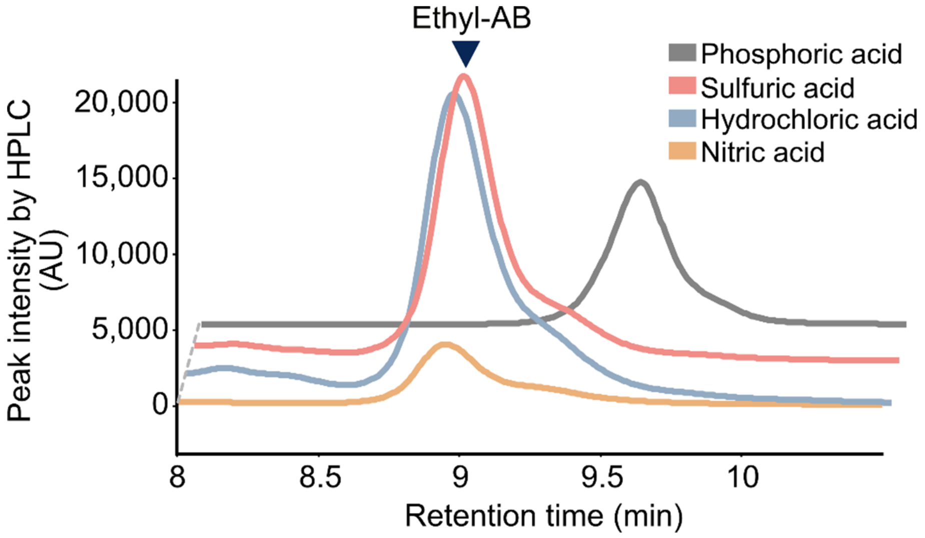

Figure S2. HPLC analysis of the reaction products of agarose alcoholysis using various acid catalysts to produce ethyl-AB. Acid-catalyzed alcoholysis was performed on 2% (w/w) agarose at 70°C for 18 h using 25 mM phosphoric acid, sulfuric acid, hydrochloric acid, or nitric acid as acid catalysts.

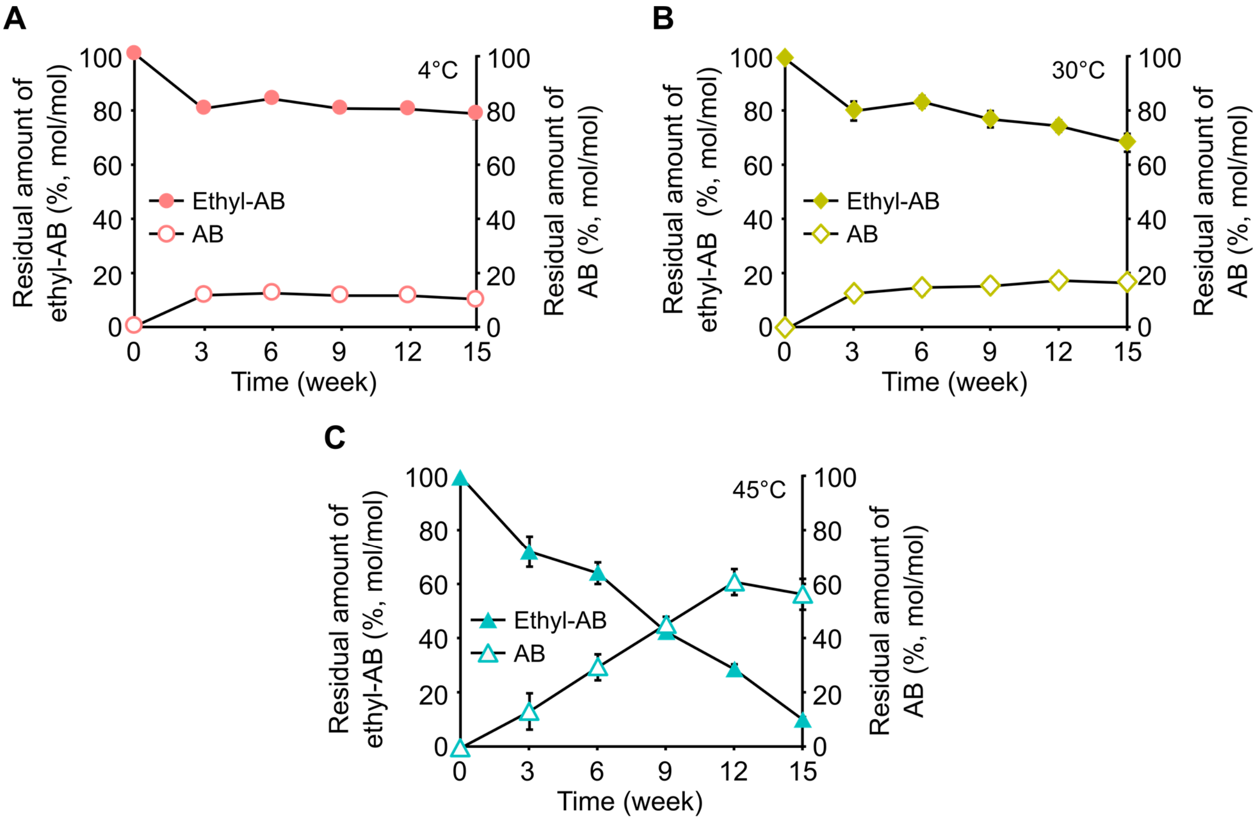

Figure S3. Thermal stability of ethyl-AB and AB as measured by their residual amounts for 15 weeks at three different temperatures. Ethyl-AB was found to be converted to AB when incubated at (**A**) 4°C, (**B**) 30°C, and (**C**) 45°C. Residual amounts of ethyl-AB and AB were measured using HPLC. All data represent the means and standard deviation of three independent replicates.
